# Supplementary material for: Elucidating the Genetic Relationships on the Original Old Sicilian Triticum Spp. Collection by SNP Genotyping
Source: Int J Mol Sci. 2022 Nov 2;23(21):13378. doi: 10.3390/ijms232113378 (PMC9694989; doi:10.3390/ijms232113378)
Supplement: Supplementary file 1 [file ijms-23-13378-s001.zip › Fiore_et_al_supplementary_material_IJMS/Fiore_et_al_supplementary_material_IJMS.pdf]

Supplementary material
Figures

Figure S1 Principal Coordinates Analysis (PCoA) of whole wheat germplasm. The varieties were highlighted

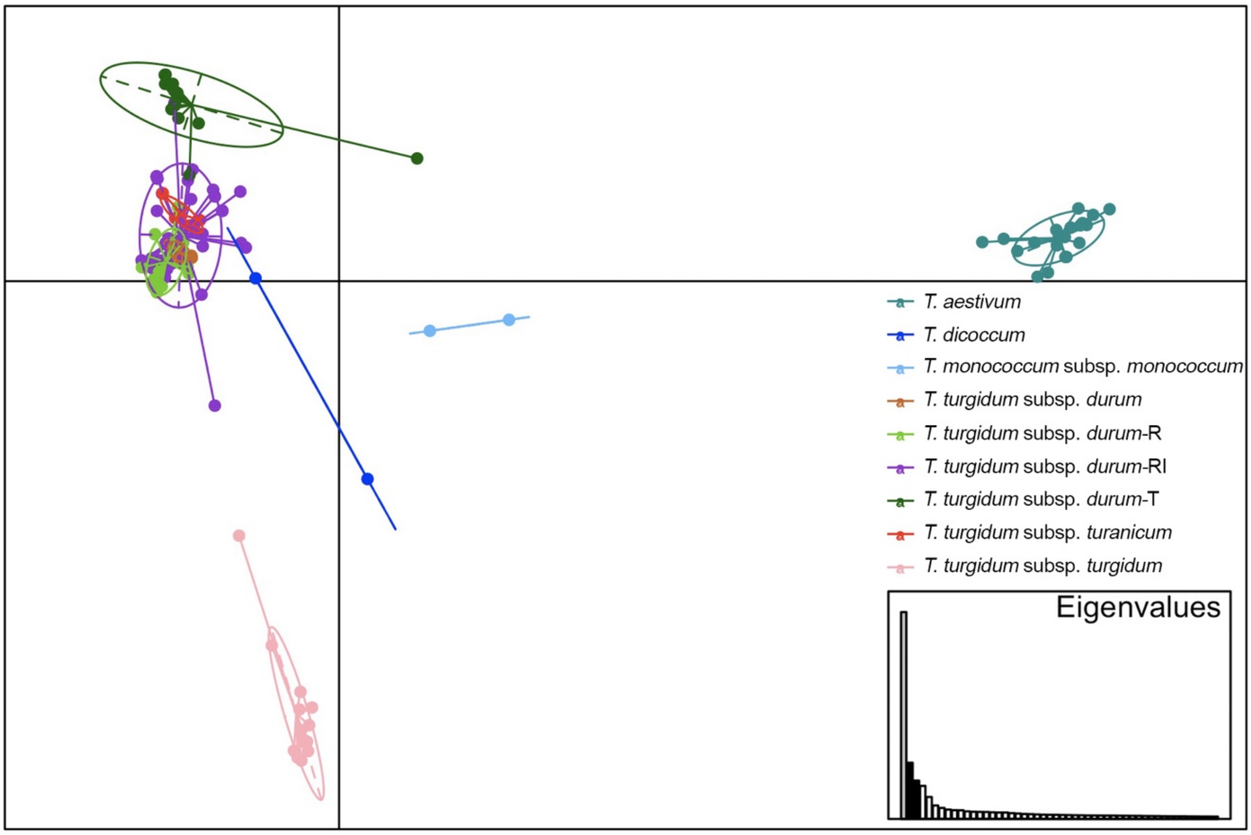

based on their species and membership group: *T. aestivum* (cyan); *T. dicococcum* (blue); *T. monococcum* subsp. *monococcum* (light blue); *T. turgidum* subsp. *durum* (brown); *T. turgidum* subsp. *durum*-R ('Russello', light green); *T. turgidum* subsp. *durum*-RI ('Russello Ibleo', violet); *T. turgidum* subsp. *durum*-T ('Timilia', dark green); *T. turgidum* subsp. *turanicum* (red); *T. turgidum* subsp. *turgidum.* PCoA1 ≈ 31% of genetic variance; PCoA2 ≈ 10% of genetic variance

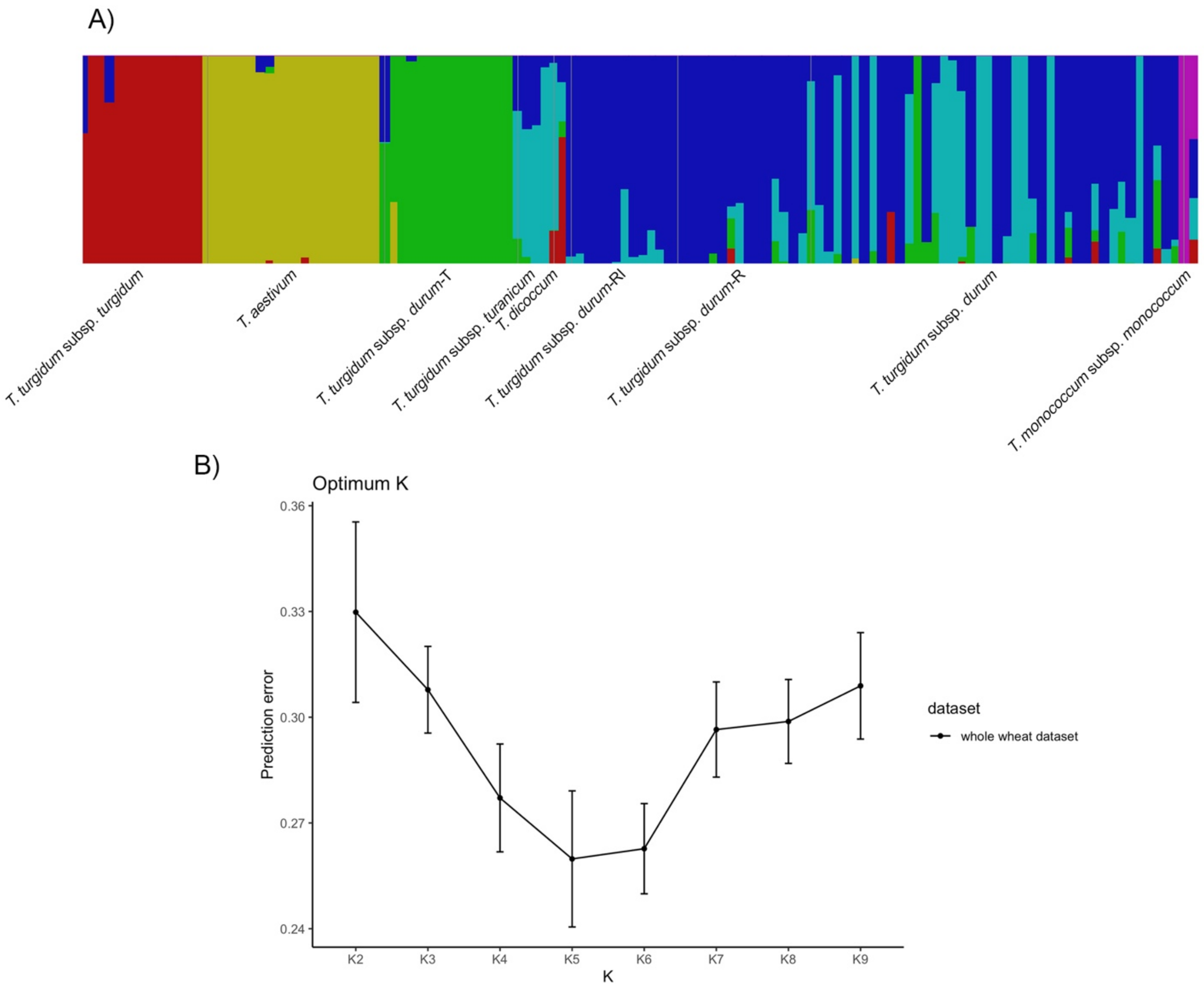

Figure S2 A) Barplot of the posterior probability for *K* = 6 (the value obtained as the model components used to explain structure in data) evaluated by the algorithm for multiple choices *chooseK.py*. **B)** The fitting number of model complexity explaining the structure of the whole germplasm investigated (126 varieties) was evaluated for *K* values ranging from 1 to 9. In addition to the multiple choices *chooseK.py* algorithm, to extract the optimum *K* within the range, the prediction error for each *K* was also computed by the cross validation (--*cv*) function. The lowest model complexity above which prediction errors do not vary significantly is considered the best one, therefore *K*=5 was chosen.

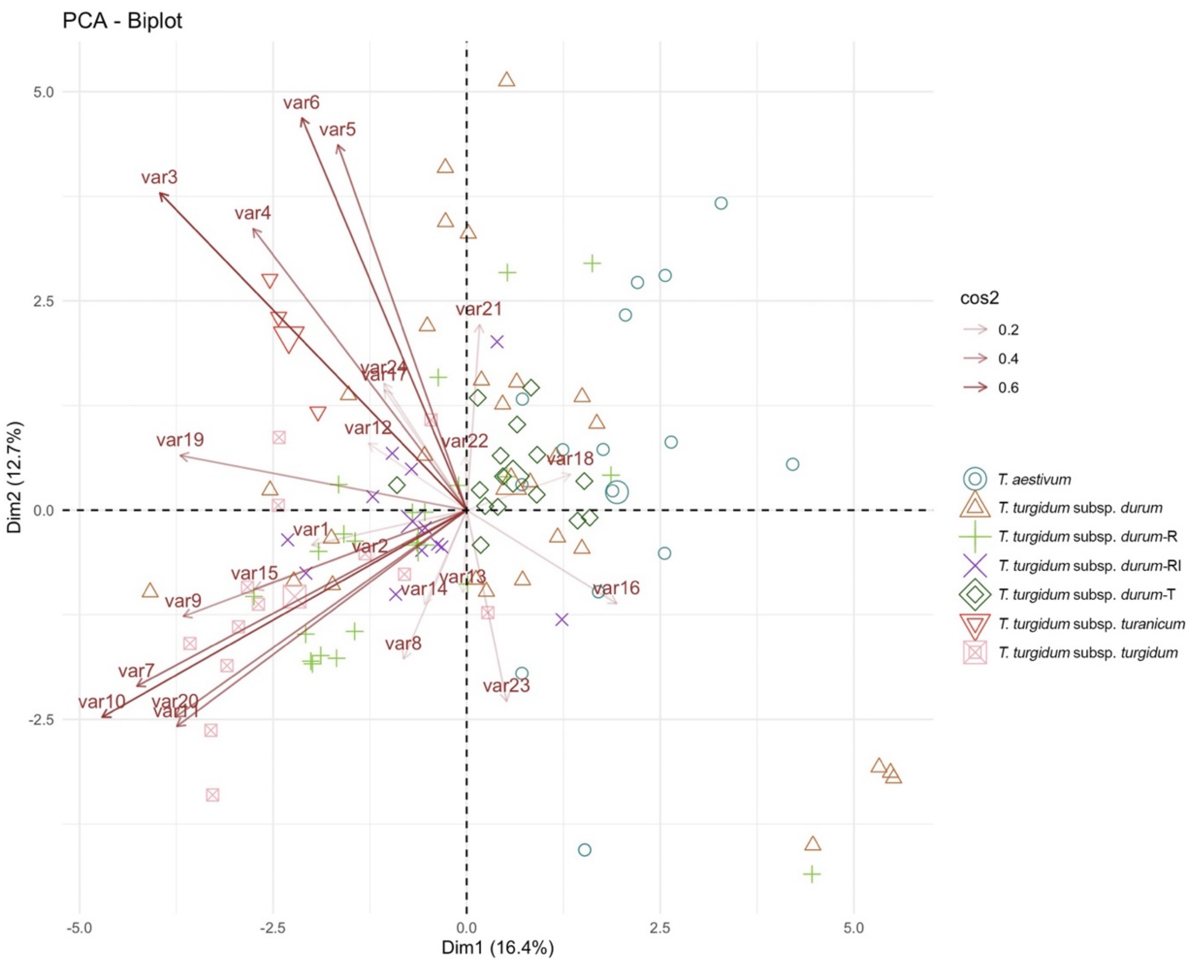

Figure S3 Principal Component Analysis (PCA) of the morphological traits evaluated for a subset (110) of varieties investigated (Table S3). Samples were organized in seven main groups: (1) *T. aestivum* (cyan); (2) *T. turgidum* subsp. *durum* (brown); (3) *T. turgidum* subsp. *durum*-R ('Russello', light green); (4) *T. turgidum* subsp. *durum*-RI ('Russello Ibleo', violet); (5) *T. turgidum* subsp. *durum*-T ('Timilia', dark green); (6) *T. turgidum* subsp. *turanicum* (red); and (7) *T. turgidum* subsp. *turgidum*. Traits associated ("var"; Table S2) with the discrimination of the samples are indicated in the plot, underlining their significance values (0.2 < cos2 < 0.6). The medians for each group were also visualized.

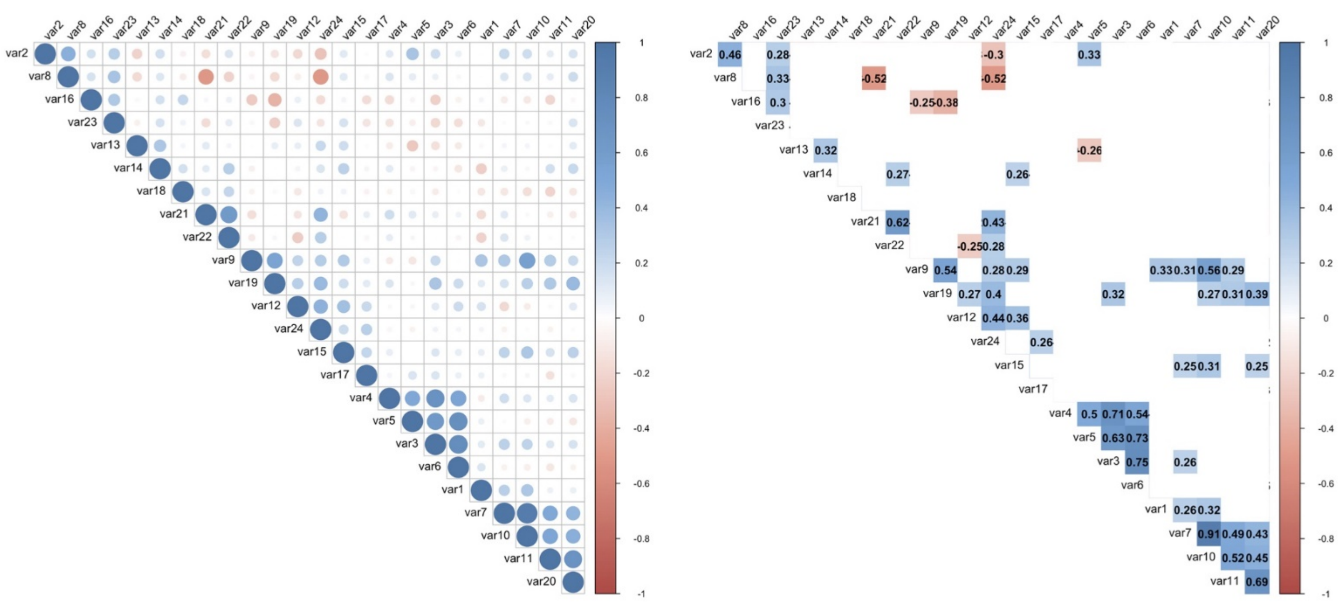

Figure S4 Person correlation matrix of 24 morphological traits. Positive correlations are displayed in blue and negative correlations in red color. Color intensity and the circle sizes are proportional to the correlation coefficients. Variables with significant correlations ($p < 0.01$) are highlighted in the plot on the right.

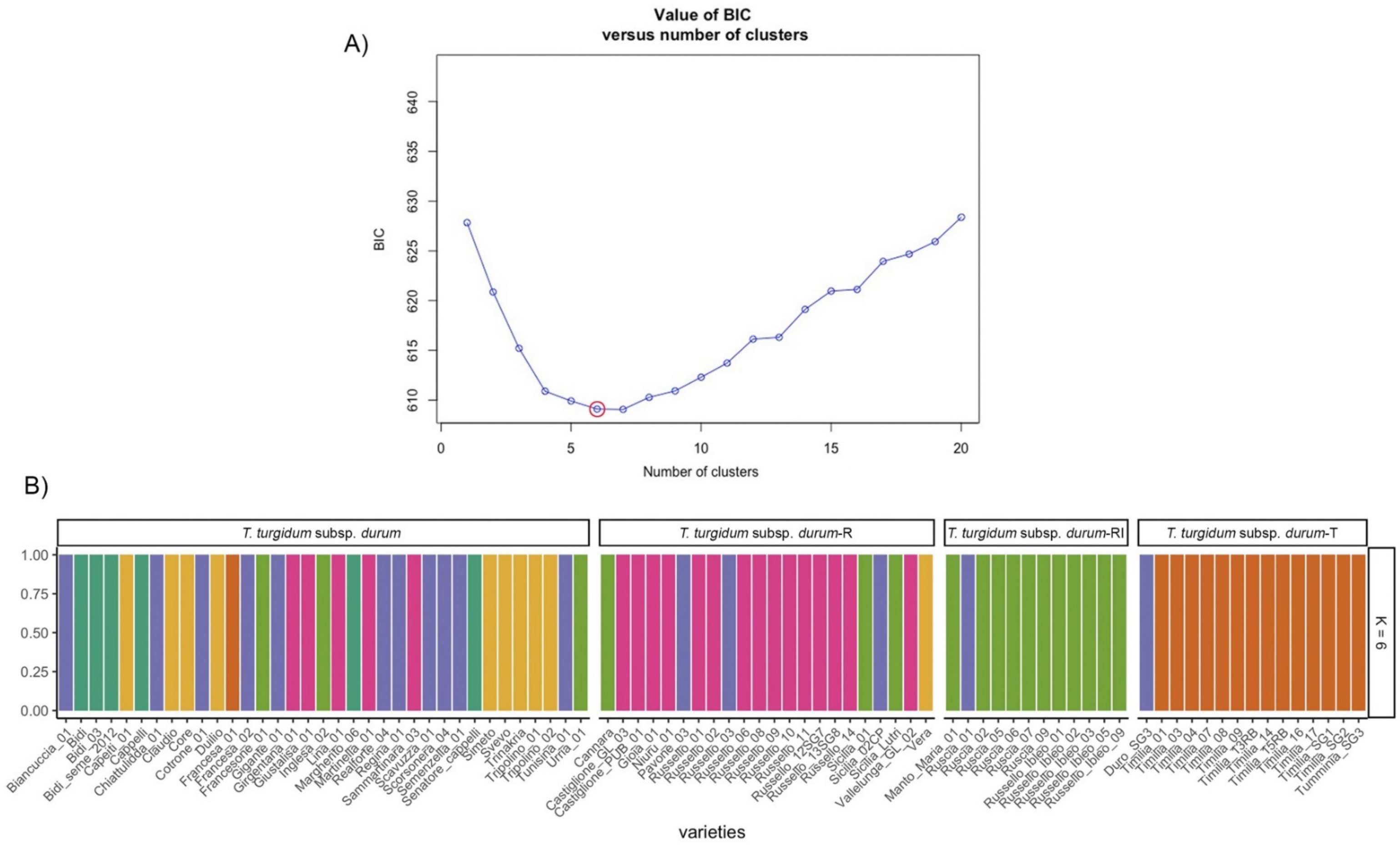

Figure S5 A) BIC (Bayesian Information Criterion) evaluated through DAPC approach for varieties belonging to *T. turgidum* subsp *durum*. BIC approach was used to infer the *K*-means clustering within the *T. turgidum* subsp. *durum* subset (*K*=6). **B)** Barplot of the posterior probability for the best *K* (6) of group assignment for each sample belonging to the subset.

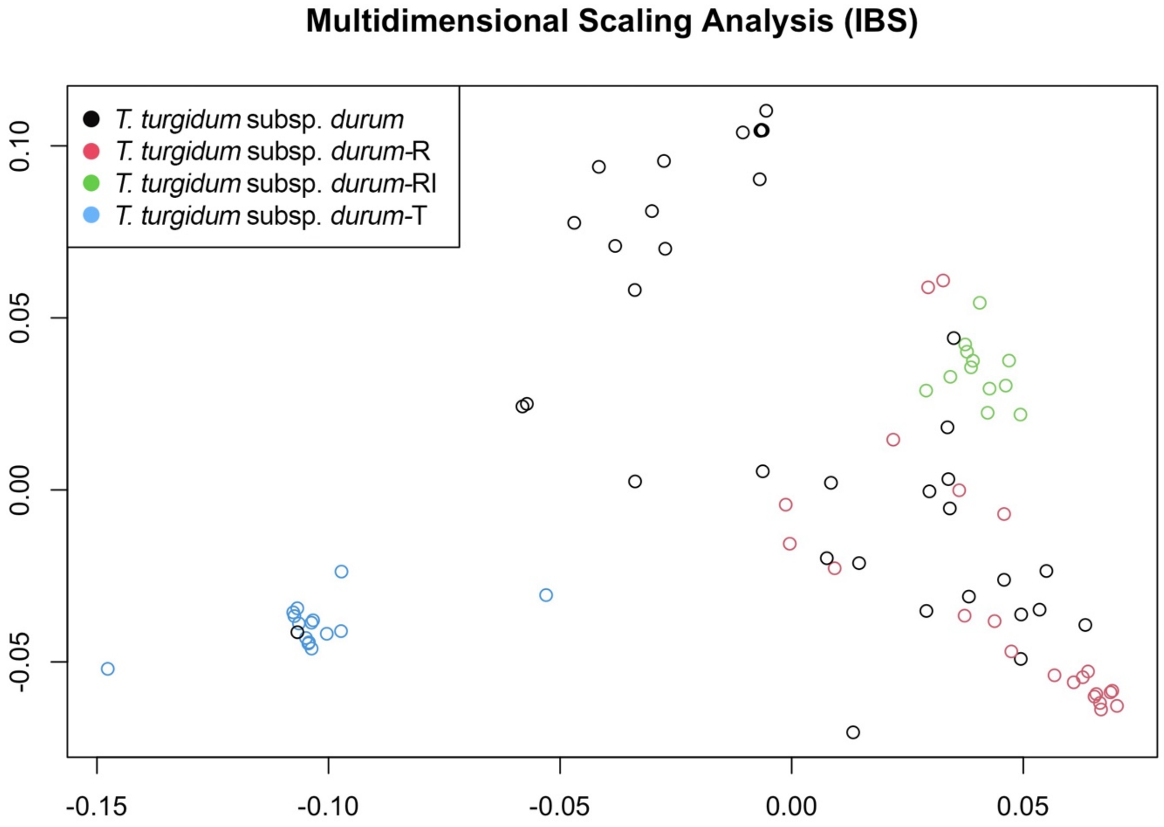

Figure S6 MDS scatterplot based on the IBS matrix of the *T. turgidum* subsp. *durum* subset. The different groups (Tables S1) were highlighted with different colors: *T. turgidum* subsp *durum* (black), *T. turgidum* subsp *durum*-R ('Russell'o; red), *T. turgidum* subsp *durum*-RI ('Russello Ibleo'; light green), and *T. turgidum* subsp *durum*-T ('Timilia'; light blue).

Tables

Table S1 SNP profiles (20,899 high-quality SNPs filtered) of samples investigated in the present work (126 varieties). The table is in .raw format.

Table S2 List of the 126 accessions belonged to *Triticum* species and subspecies analyzed by SNP markers. For each accession is reported: accession name, species, passport information and seed sources.

Table S3 Pairwise fixation index (*Fst*) values among the wheat species and groups investigated.

	T. turgidum subsp. *durum*	*T. turgidum* subsp *turgidum*	*T. turgidum* subsp. *durum*-T	*T. turgidum* subsp. *durum*-RI	*T. turgidum* subsp. *durum*-R	*T. aestivum*	*T. dicoccum*	*T. turgidum.* subsp. *turanicum*	*T. monococcum* subsp. *monococcum*
T. turgidum subsp. *durum*	-								
T. turgidum subsp *turgidum*	0.118	-							
T. turgidum subsp. *durum*-T	0.070	0.243	-						
T. turgidum subsp. *durum*-RI	0.044	0.203	0.156	-					
T. turgidum subsp. *durum*-R	0.025	0.183	0.133	0.086	-				
T. aestivum	0.238	0.238	0.252	0.232	0.243	-			
T. dicoccum	0.028	0.102	0.110	0.114	0.090	0.076	-		
T. turgidum. subsp. *turanicum*	0.026	0.178	0.122	0.117	0.099	0.142	0.226	-	
T. monococcum subsp. *monococcum*	0.046	0.149	0.147	0.161	0.129	0.068	0.292	0.300	-

Table S4 Membership group assigned before and after genetic and morphological evaluation for the whole germplasm collection (126). The probability of posterior membership for each sample included in the present study were reported. The varieties with a new membership group after genetic and morphological evaluation were underlined. The varieties belonging to the T. turgidum subsp. durum subset (84) used for the following analysis were in bold. R: 'Russello'; RI: 'Russello Ibleo'; T: 'Timilia'.

Table S5: Morphological traits evaluated for a subset (110) of varieties investigated. In the table, the species/group for each variety was highlighted. The code ("var") reported in Figure 3 and Figure S3 for each trait was also included. R: 'Russello'; RI: 'Russello Ibleo'; T: 'Timilia'.
